# Composition of trace residues from the contents of 11^th^–12^th^ century sphero-conical vessels from Jerusalem

**DOI:** 10.1371/journal.pone.0267350

**Published:** 2022-04-25

**Authors:** Carney D. Matheson, Cory R. Vickruck, Chris J. McEvoy, Kim K. Vernon, Robert Mason

**Affiliations:** 1 Department of Anthropology, Lakehead University, Thunder Bay, Ontario, Canada; 2 Department of Biology, Lakehead University, Thunder Bay, Ontario, Canada; 3 Lakehead University Molecular, Archaeochemistry and residue Services (LUMARS), Lakehead University, Thunder Bay, Ontario, Canada; 4 School of Environment and Science, Griffith University, Nathan, Queensland, Australia; 5 Royal Ontario Museum, Toronto, Ontario, Canada; New York State Museum, UNITED STATES

## Abstract

The residues from the internal surface of four archaeological ceramic sherds, excavated from the Armenian Gardens, Jerusalem were analysed to characterise the contents of the original vessel. The sherds derive from four small, thick-walled, sphero-conical vessels recovered from a destruction layer, dating between the 11th and 12th century, Jerusalem. The residue has been analysed using light microscopy, biochemical characterisation, gas chromatography mass spectroscopy, inductively coupled plasma atomic emission spectroscopy and cold vapour atomic fluorescence spectrometry. This analysis established the presence of various compounds including fatty acids and notable levels of mercury, sulphur, aluminium, potassium, magnesium, nitrates and phosphorous. The contents and probable functions of the four vessels were characterised from the residues on these sherds as different from each other, reflecting their different decoration, manufacture and ceramic typologies. One of these vessels contains residue that indicate the vessel held oils. The residue of the second vessel is consistent with either scented materials or medicinal contents, while a third probably contained medicinal material. The unique fourth sherd is from a stoneware sphero-conical vessel with very thick walls, no decoration and the residue supports the possibility it was used for the storage of chemicals or may have held the chemical ingredients for an explosive device, consistent with a medieval grenade. This residue analysis of Mamluk sphero-conical vessels provides insight into luxury items, medicines, technology and trade in medieval Jerusalem.

## Introduction

Ceramic vessels with conical bases and spheroidal bodies, ‘sphero-conical vessels’, have been found in large numbers in many varied archaeological contexts throughout the Middle East from the 9^th^ to 15^th^ century. The diversity of manufacture, decoration, vessel morphology, and their broad distribution across the Middle East, from Egypt to Central Asia, suggests that the basic vessel design was used for a wide range of functions and contents [1, 2]. Residue analysis has the potential to provide evidence that would allow us to characterise the contents of these vessels and enhance our understanding of luxury items, medicines, technology and trade in the medieval Middle East. Apart from their characteristic short neck, narrow opening, conical bases and spheroidal bodies, the size of these sphero-conical vessels varies from a few centimetres to over 20 cm in diameter with a wall thickness that varies from only a few millimetres to over 15 mm thick. They have been manufactured using a wide range of firing temperatures from low temperature firing to a specialised very high temperature firing that produces stoneware. Some vessels have been fired at temperatures in excess of 1300° C, higher than even the finest stoneware ceramics made in the Middle East [3, 4]. The ceramics are finished with an extensive range of coloured glazes coating external surfaces or left unglazed. Vessels are decorated using a wide range of styles from simple geometric patterns to complex figures; images, including zoomorphic shaped vessels; and others are undecorated. Sphero-conical vessels have been stamped with the name of the potter, owner or inscribed with phrases have been found, which has supported one interpretation of their use as drinking vessels or a beer gourd [5].

The diversity in sphero-conical vessels—design, manufacture, shape and decoration; is found in all of the regions throughout the Middle East where sphero-conical vessels have been recovered. Examples of the diversity of these vessels are displayed in museum collections all over the world [4, 6, 7] and several ceramic typologies (Table 1) have been developed to attempt to categorise that diversity [1, 2, 4, 8]. Savage-Smith [1] has categorised these vessels into ten types; Stănică and Szmoniewski [4], into four groups with three types and numerous subtypes; Vezzoli [2] into three types; while Sharvit [8] has just two types (Table 1). The Vezzoli [2] typology can differentiate the sherds used in this research and was developed for the sphero-conical vessels from Baalbek, which show a broad range of shapes, decorative schemes and surface treatments [2]. The Vezzoli Type 1 is a hard body with a range of colours (grey, brown and red), highly fired with little evidence of glazing but some surface treatments, a rounded and thickened rim, narrow opening (3–10 mm), thick walls (8–16 mm), with a plain or pinecone patterned surface or incised marks. Type 2 is overfired, with a wider range of shapes and decorative patterns but basically more spherical, coloured beige or yellow to grey with a rounded and thick rim, narrow opening (4 mm) and thick walls (10–15 mm). Some have inscriptions that have been inscribed before and after firing. Type 3 is porous with standard firing, red to orange colours and may have a wide variety of decoration including glazing and a gritty ceramic texture.

**Table 1:** The previous classification systems for sphero-conical vessels.

Author	Classification	Description
Savage-Smith [1]	Type 1 Spherical earthenware vessels	Spherical in shape with no point at the bottom, large aperture, sparse decorations, with possible inscriptions, less dense matrix, possibly used as drinking vessels.
	Type 2 Pear-shaped earthenware vessel	Rounded bottoms, little decorations, less dense matrix, possibly drinking vessels.
	Type 3 Parsnip shaped earthenware vessels	Pointed bottoms, bulging sides or shoulders, concentric rings around the top decreasing in size to the opening, no recessed collar, dense matrix.
	Type 4 Dense, unglazed earthenware	Sphero-conical shape pointed at the bottom, thick walls, dark grey, grey-green and brown in colour, dense matrix well levigated and highly fired at high temperatures, impervious to liquids and can withstand high pressures, heavy, small volume, tear shaped embossing, maybe used as plumb bobs.
	Type 5 Conical vessel of dense earthenware	Broad thick neck, dense matrix, no recessed collar, incised vertical lines dividing surface into eight panels, decorated with stamped features like scales or feathers, possibly used as aeolipiles.
	Type 6 Conical porous earthenware vessel with elaborate design	Recessed collars, made from two pieces pressed into a mould and joined at the shoulder, some have decorations of serpents., light rings incised at the bottom, sometimes have benedictory inscriptions, less dense matrix, used for holding rose-water or beverage.
	Type 7 Glazed earthenware	Glazed surface, some have benedictory inscriptions, used for holding perfume or essential oils.
	Type 8 Thick-walled glass objects	Heavy, strong walls, recessed collars, knob like necks, possibly used as plumb bobs
	Type 9 Thin-walled glass vessels with decoration	Thin walls, wide opening, usually without recessed collars, variation in size, shape and decoration, some have handles attached, decoration maybe cut, applied or moulded, used as containers for valuable liquids such as perfumes, essential oils or mercury.
	Type 10 Thin-walled glass vessels without decoration	Thin walls, wide straight necks, easy to break, used as boiling vessels, curcubit or alchemical apparatus.
Sharvit [8]	Type A Egyptian sphero-conical vessels	Dark to brownish grey, hard-fired clay, moulded with carved decorations, vertical narrow double grooves and scale patterns over the body, small neck (3cm), narrow opening (6mm), circumscribed ring at the shoulder.
	Type B Syrian sphero-conical vessel	Thick walls, some are large and heavy, fine grained, yellowish colour, hard fired, soft enough to scratch with a knife, carved, incised, stamped or affixed decorations.
Stănică and Szmoniewski^a^ [4]	Group I	Handmade on wheel, some asymmetrical, horizontally incised groove, can be very fine clay with a greenish grey glimmer, greenware of unburnt clay or stoneware with hard fine-grained matrix,
	Group II	Clay, yellowish brown, large amounts of mica, necks are deeply trimmed.
	Group III	Hard fired, heat resistant, decorated usually with imprinting rosettes, contain mica and kaolinite.
	Group IV–subtype A	Dark grey shade with mica and kaolin, no decorations.
	Group IV–subtype B	Sphero-conical shape, dark almost black clay, glazed.
	Type I	Undecorated high quality, ash-grey colour clay.
	Type II—A	Decorated with oval imprints, rosettes, vertical engraved grooves.
	Type II—B	Decorated with oval imprints, rosettes, three vertical engraved grooves.
	Type II—C	Decorated with oval imprints, rosettes, wavy lines and horizontal grooves.
	Type II—D	Decorated with oval imprints, two rosettes, two wavy lines and four horizontal rills.
	Type III—A	Oval imprints or seals in one line around upper part.
	Type III -B	Oval imprints or seals in irregularly scattered flanked by two vertical rills.
	Type III—C	Oval imprints or seals all over the body, with three vertical lines.
	Type III—D	Oval imprints or seals in a triangle on the lower part, rills and lamellas in the upper part.
Vezzoli [2]	Type 1a	Hard body, dark grey-black, brown-purple in colour, fired at high temperatures, very dense almost vitrified, decorated with pine cone patterns, round and thickened rim, narrow opening (3-10mm), thick walls (8-16mm). pointed base, some can have deep incisions.
	Type 1b	Hard body, dark grey-black, brown-purple in colour, fired at high temperatures, very dense almost vitrified, decorated with pine cone patterns, round and thickened rim, narrow opening (3-8mm), thick walls (6-10mm). pointed base, elegant with pronounced shoulder, small rim, some can have deep incisions and undecorated.
	Type 2a	Beige-yellow to a grey-green colour, can be overfired with black core, many shapes and patterns with elongated bodies, moulded, impressed with pinecone patterns or incised decorations, rounded with thick rim narrow opening (4mm) and thick walls (10-15mm), about 14 cm high, thick base, some with inscriptions.
	Type 2b	Beige-yellow to a grey-green colour, can be overfired with black core, many shapes with moulded, impressed and incised decorations, rounded shape, small conical base, occasionally moulded shoulders with a wide range of decorations moulded, impressed, incised decorations usually located on the upper part, small rounded rim, opening 6-8mm, walls 6-15mm thick bases.
	Type 3	Porous body, less dense clay, red or orange colour, lower temperature firing, unglazed or glazed (turquoise or green), plain or decorated, with pinecone patterns common.

^a^ The classification system by this author is in two forms. They are grouped by manufacture and shape and typed based on decorations. Therefore, each sphero-conical vessel will have a group and type assigned to them.

There have been many uses proposed for sphero-conical vessels. The simple utilitarian uses based on their size, weight and shape include a plumb bob [1], a loom weight and an architectural feature [9]. If considered a vessel the proposed uses include liquid sprinkler [9], fire starter [10, 11], fire blower [12], aeolipile [9, 12–14], lamp [4, 15], smoking pipe [7, 16], alchemical vessel [7, 13, 16, 17] or parts of a distillation apparatus [17, 18]. From historical documents there is support for thick-walled vessels being used in warfare as grenades [9, 12, 19, 20]. However, the most common function proposed for sphero-conical vessels is a container. Suggested vessel contents include liquids for human consumption including wine, honey [9], beer [5] and medicines [9, 13, 21]. Alternatively, valuable liquids have also been proposed like mercury [9, 22], oil [6], rosewater [7, 23], scented oil [6], holy water [9], ink [1], scented water [24] and perfume [9, 25]. Few of these proposed uses have been tested or confirmed.

There are very few analyses of residue within these sphero-conical vessels to confirm any of these purported uses (Table 2. The earliest attempt was made by Maskelyn, in 1871 who identified mercury on vessels from Saïda, and this was repeated by Vysotskii in 1908 on vessels from Old Krym and again in 1914 by Lenz on two vessels one from Kazan and the other from Moscow [9]. All of these researchers came to the conclusion that their vessels were used to store and transport mercury. Mercier [26] analysed residues from sphero-conical vessels from Fustat (Old Cairo), reported as grenades to have been used by the Arabs against the Crusaders in AD 1168 (CE) and identified potassium nitrate and sulphur, typical of explosive material, consistent with the proposed use as incendiary or explosive weapons. A study by Brosh [10] on the contents of sphero-conical vessels of unknown provenance, identified iron and proposed this was from iron pyrite which could have served as a fire starter. A study on a sphero-conical vessel from Bolgar, Russia, identified resin acids, initially interpreted as part of distillation apparatus [18], later reinterpreted as a medicinal kit [21]. Finally, fats and oils were characterised on sphero-conical vessels from Armenia that were proposed to be perfume or medicinal containers [27]. However, considering the diverse nature of sphero-conical vessels, these limited examinations offer only a few potential uses from a potentially unrepresentative small set of vessels. The purpose of this research is to characterise residues from four sphero-conical vessels of different styles to help understand the function of these vessels.

**Table 2:** Previous analysis and proposed uses of sphero-conical vessels.

Analyst	Year	Location	Identified	Proposed use	Ref
Maskelyn	1871	Saïda	Mercury	Store and transport mercury	[9]
Vysotskii	1908	Old Krym	Mercury	Store and transport mercury	[9]
Lenz	1914	Kazan	Mercury	Store and transport mercury	[9]
Lenz	1914	Moscow	Mercury	Store and transport mercury	[9]
Mercier	1937	Fustat ()	Potassium nitrate, sulphur	Grenade	[26]
Brosch	1980	unknown	Iron	Fire starter (Iron pyrite)	[10]
Pozhidaev	2016	Bolgar	Resin acids	Distillation apparatus later	[18]
				Medicinal kit	[21]
Barnard	2016	Dvin (4)	Fats and oils	Perfume or medicinal	[27]

## Materials and methods

### Samples

The sherds investigated in this study were excavated in Jerusalem between 1961 and 1967, from the Armenian Garden. This area includes the site of the Crusader royal palace [28–30]. According to Tushingham [31] they date to the 11-12^th^ century from Mamluk contexts. These sherds have been catalogued in the Royal Ontario Museum collections (Table 3). Using the Vezzoli [2] typology, sherd 737 (ROM.968.353.737_1) would be type 1, sherd 741 (ROM.968.353.741_1) would be classed as type 3, while both sherds 742 (ROM.968.353.742_1) and 744 (ROM.968.353.744_1) would be type 2. The Sharvit [8] typology with just two categories is difficult to apply. Likewise, it is difficult to categorise these sherds using the Savage-Smith [1] typology since they are missing the required diagnostic characteristics. None of the sherds received conservation or treatment after excavation except being lightly brushed and washed with water. All four ceramic sherds have traces of residue that could be described as amorphous i.e. without any morphological structure or features that could be used for microscopic identification. Permission to analyse these items was provided by the Royal Ontario Museum.

**Table 3:** The details and description of each sherd and residue.

Sherd	Catalogue number	Excavation	Description	Residue
737	ROM.968.353.737_1^a^	Excavated from the destruction debris above the medieval surface^a^	A dense grey stoneware, the same grey surface inside and out^b^	Traces of amorphous residue, thin, homogeneous in texture, mixed light brown and dark grey in colour and accumulated on the interior surface imperfections that cover about 20% of the interior surface of the sherd
741	ROM.968.353.741_1^c^	Excavated from excavation locus 250.3a^d^	An orange-buff ware with some tiny brown grits, an inner surface fired reddish brown except where some dark slip has entered from the top and the exterior has a poor quality black slip^e^	Traces of amorphous residue, dark brown almost black coloured residue of homogeneous texture that was found on a small portion of the interior surface
742	ROM.968.353.742_1^f^	Excavated from the destruction debris above the medieval surfaces at excavation locus 1.4c^d^	A greyish green ware with a few minute white grits, the inside surface is pale green and the outer surface is dark green^e^	Traces of amorphous residue, dark brown residue inside the opening, but its extent is difficult to determine as the opening is very narrow
744	ROM.968.353.744_1^g^	Excavated from the destruction debris above the medieval surface at excavation locus 50.2c^d^	A dense greenish ware with a pale grey surface on the interior side^e^	Traces of amorphous residue, thin, light brown, homogeneous and found on a small portion of the interior surface

^a^Tushingham [31].

^b^Authors description as there is no description in the ROM catalogue.

^c^Tushingham [31], pg. 397, fig 45 #9.

^d^Tushingham [31], pg. 150.

^e^Tushingham [31], pg. 341.

^f^Tushingham [31], pg. 397, fig 45 #8.

^g^Tushingham [31], pg. 397, fig 45 #10.

### Soil

Since the artefacts are from excavations conducted in the 1960s, it is not possible to obtain soil samples from the excavation units, however Tushingham [31] reports elemental analysis of a flaked piece of soil-covered material from the surface of an iron sword, excavated from the same site. There is available data on the elemental composition of soils from this area of Jerusalem which have been used for comparison [32–34].

### Residue removal

The residue was removed by applying a solution of LCMS grade ethanol, water and acetonitrile (1:1:1 v/v/v—Sigma-Aldrich). This mixture was selected for its capacity to dissolve a broad range of compounds from the unknown residue, shown to be effective at removing residue from various archaeological materials [35–37]. For sherd 742 a volume of 2.0 mL of removal solution was placed into the cylindrical opening for 30 min. For the other three artefacts, the same volume of removal solution was placed on the concave internal surface in a position that retained the solution. After 30 min the solution was removed from the surface of the sherds and placed into sterile 2.0 mL acid washed glass vials. There was no absorption of the solution into the impervious ceramic. The amount of residue removed was very small and unable to be measured.

### Biochemical tests

A variety of biochemical tests were employed in the initial analysis. These were all modified into a microchemical test suitable for residue analysis and performed in duplicate. The Bradford colorimetric test, to detect protein or peptides [38] was modified to use 5.0 μL volume of the removed residue, freeze dried until dry and mixed with 1.0 μL of the Bradford Reagent. The solution was heated at 25° C for 20 min and the reaction measured at 595 nm using the Epoch^TM^ Multi-Volume Spectrophotometer System (Biotek) using Gen 5 software with standard references. A diphenylcarbazide/copper triethanolamine (DPC/Cu-TEA) colorimetric test, for the detection of fatty acids was modified from Falholt, et al. [39]. Again, 5.0 μL of removed residue was freeze dried and resuspended in 5.0 μL of acetonitrile, then mixed with 20.0 μL of Cu-TEA and 5.0 μL of DPC and allowed to sit at 18° C for 15 min and measured at 550 nm. A colorimetric test for sulphur using pyridine was modified from Sommer [40]. A volume of 40.0 μL of pyridine was placed into a sterile 2.0 mL glass vial and 2.0 μL of 2.0 M sodium hydroxide added. The solution was heated to 70° C for 1 min. An aliquot of 5.0 μL of removed residue solution was placed into the reaction vial and the reaction measured at 580 nm. A diphenylamine colorimetric test for nitrates, was modified from Whelan [41]. Only 10.0 μL of a diphenylamine solution (10 mL water; 90 mL sulphuric acid; 0.5 g of diphenylamine) was mixed with 10.0 μL of the removed residue solution and the reaction measured at 595 nm.

### Gas chromatography—mass spectroscopy analysis

A volume of 450 μL of each removed residue solution was transferred to new sterile acid washed 2.0 mL glass autosampler vials and freeze-dried under vacuum for 8 hrs or until dry. Once dried 900 μL of acetonitrile (Sigma-Aldrich) was added to each vial. The samples were derivatised using 100 μL of BSTFA (bis(trimethylsilyl)trifluoroacetamide) with 1% TMS (trimethylchlorosilane) solution (Sigma-Aldrich) and purged with nitrogen sealed and incubated at 120° C for 30 mins (Baxter Scientific Multi-Block). The samples were analysed with GC-MS (Varian model 450 gas chromatograph coupled with a Varian model 300-MS quadrupole mass spectrometer) under the same conditions as Matheson and McCollum [37].

### Elemental analysis

Two methods were used for elemental analysis. For the Inductively Coupled Plasma Atomic Emission Spectroscopy (ICP-AES) analysis, a volume of 0.5 mL of the removed residue solution was transferred to a new sterile acid washed 2.0 mL glass autosampler vial and freeze dried under vacuum for 8 hrs or until dry. The residue was resuspended in 500 μL of water. A mixture of the water-residue solution and 1.0 mL of 98% nitric acid was heated at 180° C, for 3 hrs in an acid digestion bomb. After digestion, the water-residue-nitric acid solution was mixed with 8.5 mL of water to create a 10% nitric acid solution and analysed by ICP-AES (Varian Vista Pro Radial). The presence of mercury (Hg) in the residue was tested by Cold Vapour Atomic Fluorescence Spectrometry (CVAFS). A 1.0 mL sample of the removed residue was diluted to 10.0 mL with ultrapure LCMS grade water. The sample was preserved by adding 12 N hydrochloric acid (HCl) solution and the mercury was oxidised to Hg(II) with bromine chloride (BrCl). The sample was reduced using ammonium hydroxide-hydrochloric acid (NH_2_OH-HCl) solution to destroy any free halogens and reduced again with stannous chloride (SnCl_2_) to convert Hg(II) to volatile Hg(0). The Hg(0) was separated from the solution by purging with helium and collection onto a gold trap. The Hg(0) was thermally desorbed from the gold trap into an inert gas stream that carries the released Hg(0) to a second gold (analytical) trap. Finally, the Hg(0) was desorbed from the analytical trap into a gas stream that carries it to the cell of the CVAFS machine (Brooks Rand) for detection.

### Microscopy

After the chemical tests were performed, the residual particulate material was placed onto microscope slides and characterised using an Olympus BX51 microscope under bright field and polarised transmitted light.

### Analysis and interpretation

The data was assessed to ensure accurate identification of elements and compounds. Then, the data was evaluated to distinguish results that came from the residue as opposed to the depositional environment, from the composition of the ceramics or from contamination during excavation, storage, analysis or handling. Any result considered to be potential contamination was excluded from the interpretation. Finally, an interpretation was made based on data considered consistent and reliable.

## Results

The microscopic analysis supports the identification of a mixture of both organic and inorganic material on the internal surfaces of all the sherds. It identified a collection of inorganic crystals, burnt organic material (not charcoal), plant tissue, cellulose fibres and hair fibres. The biochemical tests resulted in the detection of fatty acids on all four of the artefacts. The Bradford test for proteins, produced weak positive results on sherds 741, 737 and 742. Both the nitrate and sulphur tests produced positive results for sherd 737. The results of the biochemical tests for fatty acids were verified using GC-MS. The positive results of the Bradford test were consistent with the amino acids detected in the GC-MS, while the positive result of the sulphur tests on sherd 737 was verified using ICP-AES. Elemental analysis by ICP-AES detected sulphur in all four samples (Table 4). However, the amount of sulphur in the residue from sherd 741 is the lowest, it is still greater than the background soil levels. The amount of sulphur detected by ICP-AES in the residues from sherds 737, 742 and 744 are all greater than that found in depositional soil and can be attributed to the residue. Although the residue from vessel 737 has the highest amount of sulphur.

**Table 4:** The ICP-AES elemental composition of each artefact (ppm).

	Al	Ca	Fe	K	Mg	Na	P	Pb	S	Si
737	24,880	513,600	16,256	11,912	59,920	14,980	14,388	2,636	142,480	73,360
741	368	8,628	412	1,988	1,192	14,544	2,616	208	12,464	708
742	1,308	26,328	1,236	508	1,456	20,620	3,148	984	51,880	3,424
744	1,900	12,796	676	ND	1,420	6,804	ND	ND	48,120	5,040
soil	13,400	101,000	7,600	3,200	5,000	16,400	2,000	200	3,200	54,200

The soil values are calculated from various sources [32–34 and unpublished data].

The results of the ICP-AES were converted into ratios between the elements within each sample for comparative purposes (Table 4). Some of the elements (As, Be, Cd, Co, Cr, Li, Mo, V and Zr) were not identified on any of the sherds while others (B, Ba, Cu, Mn, Ni, Sr, Ti and Zn) were found in low concentration in all the samples as well as the soil and were subsequently excluded. The ratios of elements Al, Ca, Fe, K, Mg, Na, P, Pb, S and Si, varied greatly between each sherd (Table 4). Interpretation was based on the assumption that any element found in an amount above the background level, including the soil, is from the archaeological residue. The results from sherd 737 indicated elevated Al, Ca, Fe, K, Mg, Na, P, Pb and S. Sherd 742 only had large amounts of Ca and Na, while sherd 744 had elevated levels of Ca, Na and S. Finally, the elemental analysis of sherd 741 does not show any elemental composition that can be attributed to the residue as it has no elemental results from the ICP-AES that is higher in quantity than the background soil. Therefore, the elemental results for sherd 741 could be attributed to soil infill from the depositional environment.

Mercury was detected on two artefacts using CVAFS (737 and 742). The results show that the extracts from sherd 742 had mercury at 4,900 ppb and sherd 737 had 13,920 ppb, ten and twenty-eight times the background soil concentrations (490 ppb) respectively.

**Fig 1:**
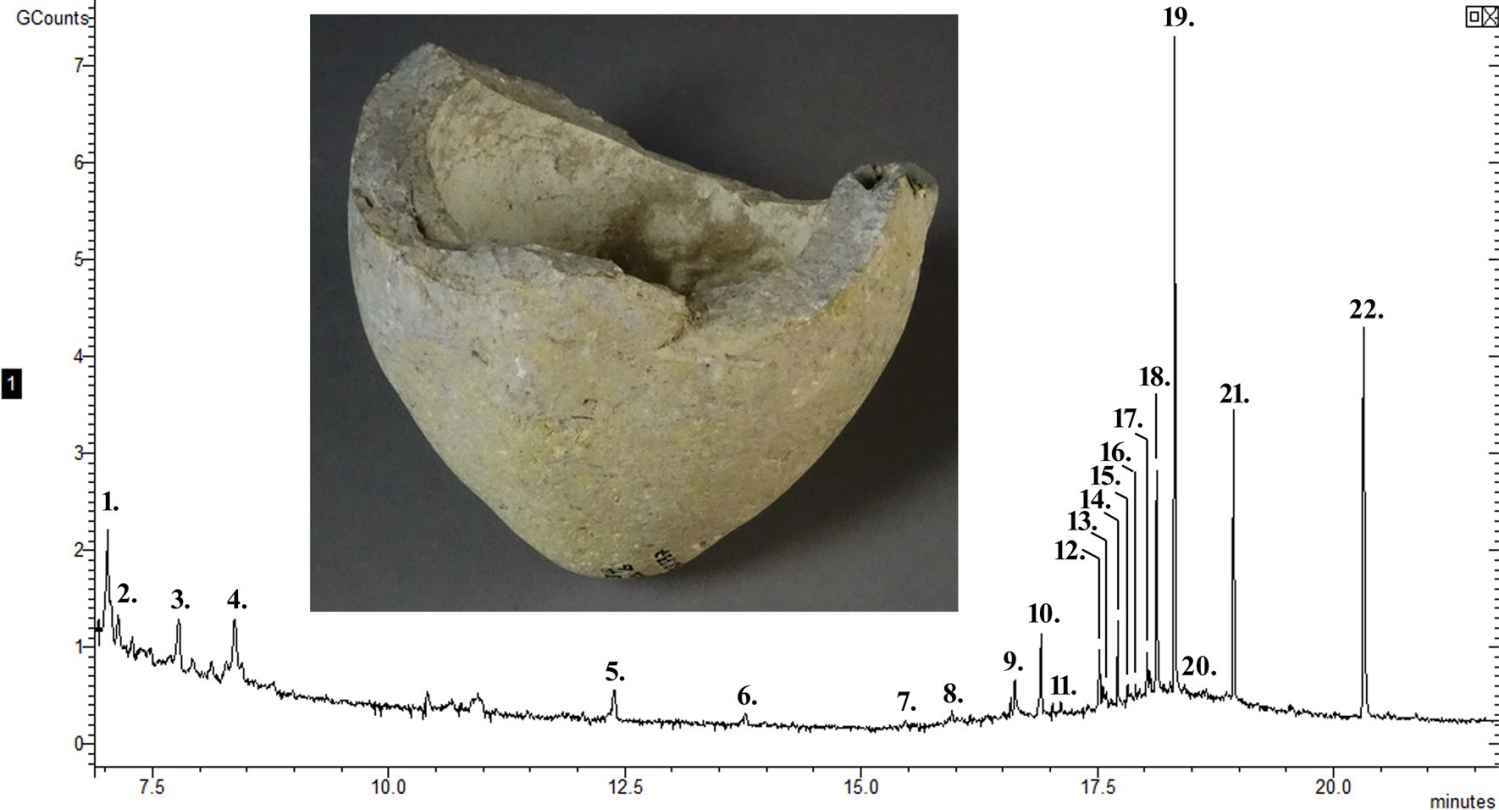
The chromatogram of the residue from sphero-conical sherd 737. The peaks identify compounds 1—glycine, 2—leucine, 3—methylmalonic acid, 4—lactic acid, 5—glycerol, 6—unidentified, 7—capric acid, 8—salicylic acid, 9—diethylphthalate, 10—lauric acid, 11—suberic acid, 12—azelaic acid, 13—carballylic acid, 14—myristic acid, 15–7-methylhexadecane, 16—pentadecylic acid, 17–1-monolinoleoylglycerol, 18—cetyl alcohol, 19—palmitic acid, 20—unidentified, 21—stearic acid, 22—phthalic acid, mono(2-ethylhexyl) ester.

**Fig 2:**
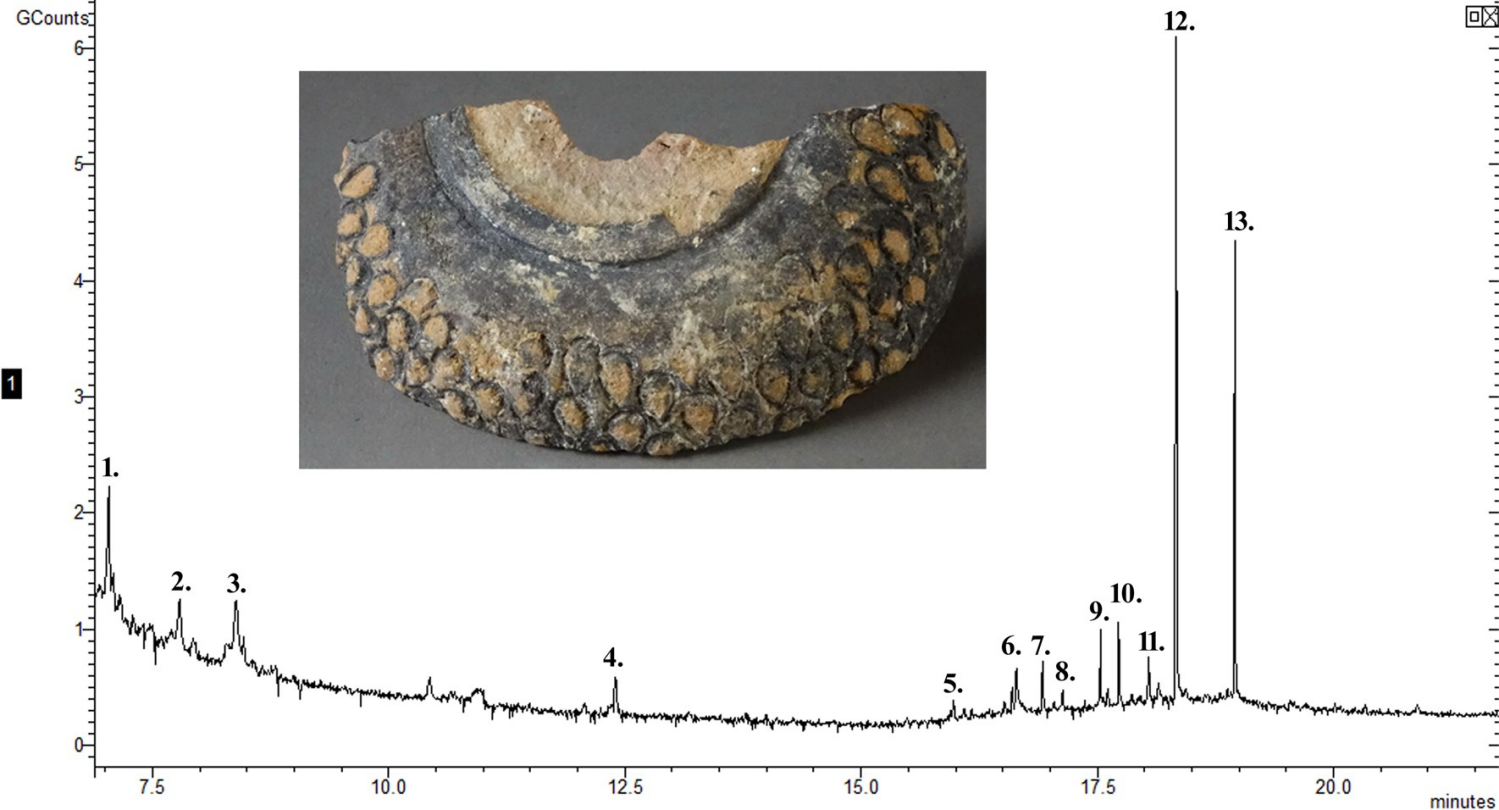
The chromatogram of the residue from sphero-conical sherd 741. The peaks identify compounds 1—alanine, 2—methylmalonic acid, 3—cyclohex-1-en-1-ol, 4—glycerol, 5—salicylic acid, 6—diethylphthalate, 7—lauric acid, 8—suberic acid, 9—azelaic acid, 10—myristic acid, 11–1-monolinoleoylglycerol, 12—palmitic acid, 13—stearic acid.

**Fig 3:**
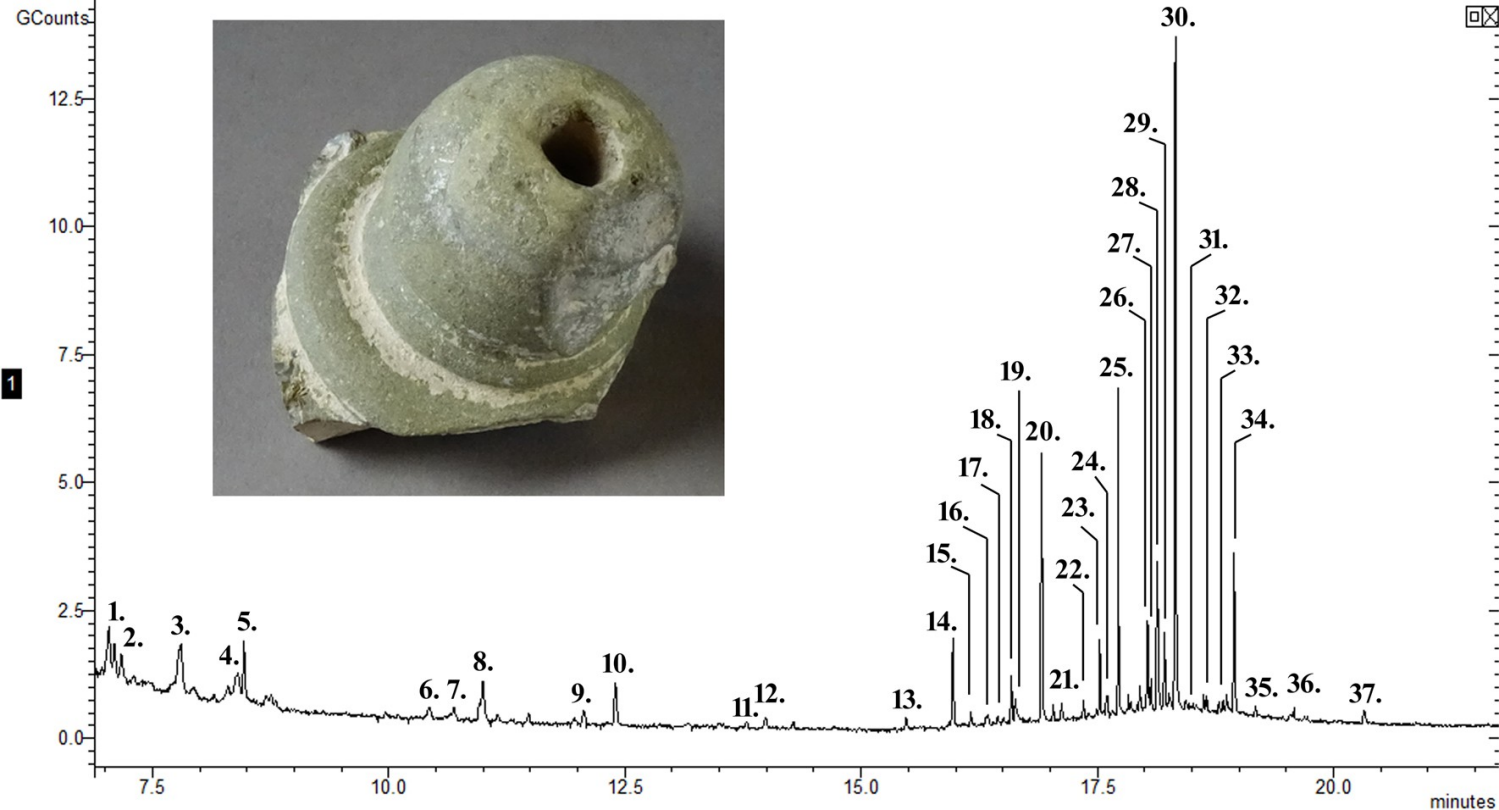
The chromatogram of the residue from sphero-conical sherd 742. The peaks identify compounds 1—alanine, 2—unidentified, 3—unidentified, 4—cyclohex-1-en-1-ol, 5—lactic acid, 6—unidentified, 7—levulinic acid, 8—enanthic acid, 9—urea, 10—glycerol, 11—glycine, 12—pelargonic acid, 13—capric acid, 14—salicylic acid, 15—undecanoic acid, 16–4-methylvaleric acid, 17–10-undecynoic acid, 18–4-methoxycinnamic acid, 19–3-hydroxybenzoic acid, 20—lauric acid, 21—suberic acid, 22—tridecylic acid, 23—xylofuranose, 24—azelaic acid, 25—myristic acid, 26—pentadecylic acid, 27—cetyl alcohol, 28—cyclohexanecarboxylic acid, 4-(1,5-dimethyl-3-oxohexyl)- methyl ester, 29—palmitoleic acid, 30—palmitic acid, 31—margaric acid, 32—hexadecanoic acid, 1,1-dimethylethyl ester, 33—oleic acid, 34—stearic acid, 35–2-methylnonadecane, 36—dehydroabietic acid, 37—phthalic acid, mono(2-ethylhexyl) ester.

**Fig 4:**
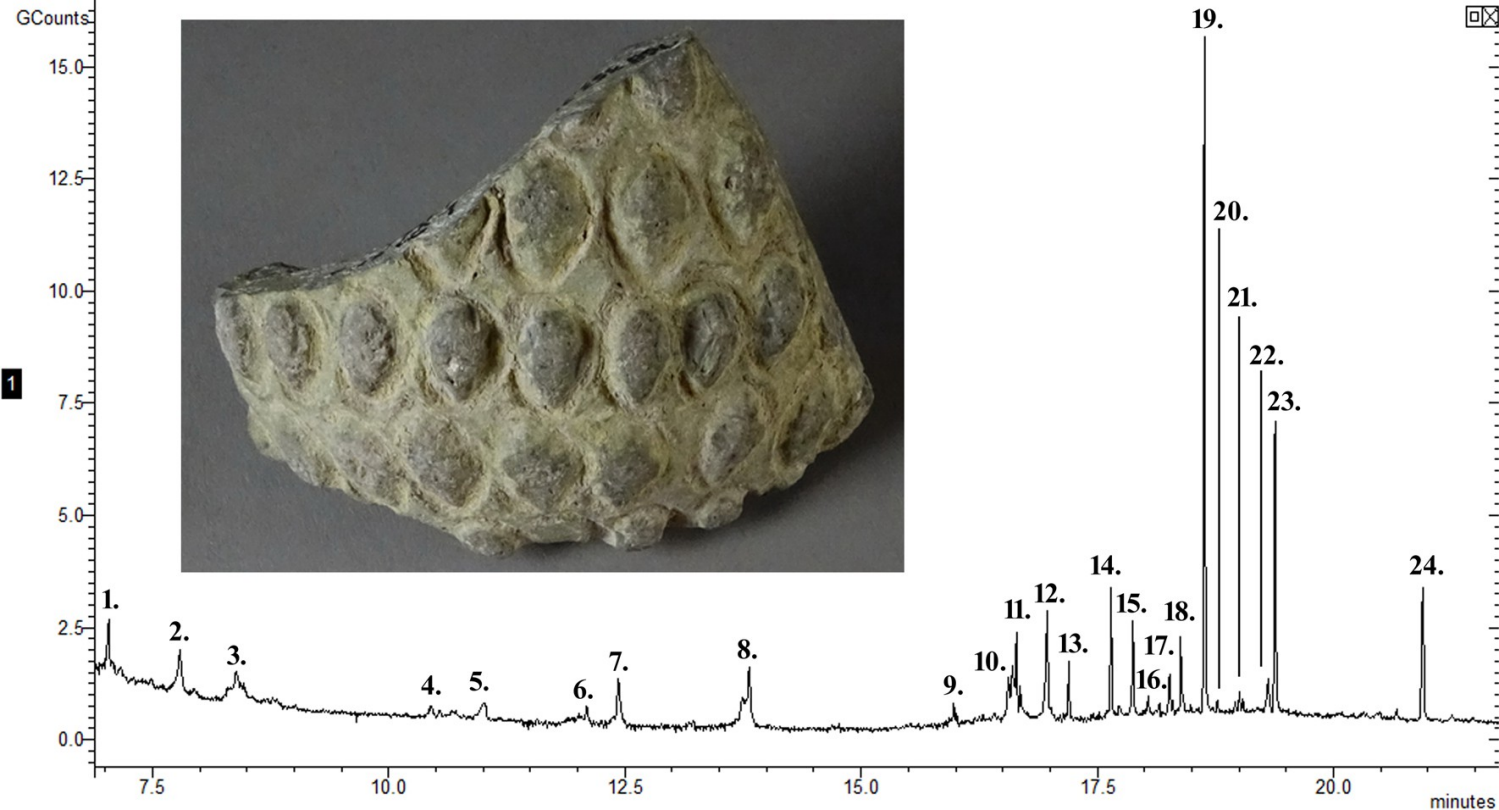
The chromatogram of the residue from sphero-conical sherd 744. The peaks identify compounds 1—methylmalonic acid, 2–2-hydroxytetradecane, 3–2-cyclohexen-1-ol, 4–2-acetylresorcinol, 5—unidentified, 6—benzoic acid, 7–2-(4-methoxyphenyl)-2-(4-hydroxyphenyl)-propane, 8–1-bromo-4-iodo-benzene, 9—adipic acid, 10–3-methoxycinnamic acid, 11–4-methoxycinnamic acid, 12—cyclohexyl, ethyl phthalate, 13—lauric acid, 14—azelaic acid, 15—myristic acid, 16—sebacic acid, 17—pentadecylic acid, 18—cetyl alcohol, 19—palmitic acid, 20—dodecanoic acid, 2,3-bis(acetyloxy)propyl ester, 21—margaric acid, 22—stearyl alcohol, 23—stearic acid, 24–17a-ethyl-3.beta.-methoxy-17a-aza-D-homoandrost-5-ene-17-one. Sample 744 was analysed at a later date than the other three samples and the column length was different resulting in a slight shift in retention times.

The GC-MS analysis identified the presence of a variety of organic compounds, from plant and animal sources, in the residues from all the sherds (Table 5). Amino acids were identified on three of the four sherds (737,741 and 742) while carbohydrates and resin acids were identified on one sherd (742). Hydrocarbons were identified on only two sherds (737 and 742). Some identified compounds were excluded from the interpretation of the data as probable contamination because they were present in the extraction solutions, were generated during the derivatisation process or were potentially environmental. Each of the chromatograms (Figs 1–4) showed varying compounds demonstrating the different composition of the residue on the inside surface of each sherd.

**Table 5:** The compounds identified in the residue from the sphero-conical vessels using GC-MS.

Chemical compounds	Artefact			
IUPAC name (common name)	737	741	742	744
**Fatty acid**				
heptanoic acid (enanthic acid)^a^^,^ ^b^			√	
nonanoic acid (pelargonic acid)^b^^,^ ^c^			√	
decanoic acid (capric acid)^a^^, c^	√		√	
undecanoic acid^c^			√	
dodecanoic acid (lauric acid)^c^	√	√	√	√
tridecanoic acid (tridecylic acid)^a^^,^ ^c^			√	
tetradecanoic acid (myristic acid)^a^^,^ ^c^	√	√	√	√
pentadecanoic acid (pentadecylic acid)^a^^,^ ^b^^,^ ^c^	√		√	√
hexadecanoic acid (palmitic acid)^a^^,^ ^b^^,^ ^c^	√	√	√	√
heptadecanoic acid (margaric acid)^a^			√	√
octadecanoic acid (stearic acid)^a^^,^ ^b^^,^ ^c^	√	√	√	√
cis-9-hexadecenoic acid (palmitoleic acid)^a^^,^ ^c^			√	
9-octadecenoic acid (oleic acid)^a^^,^ ^c^			√	
10-undecynoic acid^c^			√	
**Carboxylic acids**				
lactic acid^a^^,^^b^^,^^c^	√		√	
4-oxo-pentanoic acid (levulinic acid)^a^^,^ ^c^			√	
4-methylpentanoic acid (4-methylvaleric acid)^c^			√	
benzoic acid^c^				√
3-hydroxybenzoic acid^a^^,^ ^c^			√	
2-hydroxybenzoic acid (salicylic acid)^c^	√	√	√	
1,2,3-propanetricarboxylic acid (carballylic acid)^c^	√			
cyclohexanecarboxylic acid			√	
**Dicarboxylic acids**				
Methylpropanedioic acid (methylmalonic acid)	√	√		√
hexanedioic acid (adipic acid)^a^				√
octanedioic acid (suberic acid)^c^	√	√	√	
nonanedioic acid (azelaic acid)^a^^,^ ^b^^,^ ^c^	√	√	√	√
decanedioic acid (sebacic acid)^c^				√
**Amino acids**				
(2S)-2-aminopropanoic acid (alanine)^a^^,^ ^c^		√	√	
2-aminoacetic acid (glycine)^a^^,^ ^c^	√		√	
(2S)-2-amino-4-methylpentanoic acid (leucine)^a^^,^ ^c^	√			
**Hydrocarbons**				
7-methylhexadecane^a^^,^ ^c^	√			
2-methylnonadecane^a^^,^ ^c^			√	
3,3-diphenyl-1,2-propadiene			√	
**Carbohydrates**				
xylofuranose^c^			√	
**Esters**				
dodecanoic acid, 2,3-bis(acetyloxy)propyl ester^c^				√
hexadecanoic acid, 1,1-dimethylethyl ester^c^			√	
1-Cyclohexene-1-carboxylic acid, 4-(1,5-dimethyl-3-oxohexyl)-, methyl ester (4-(1,5-dimethyl-3-oxohexyl)-methyl ester)			√	
phthalic acid, mono(2-ethylhexyl) ester^b^	√		√	
**Alcohols**				
hexadecanol (cetyl alcohol)^a^^,^ ^c^	√		√	√
octadecanol (stearyl alcohol)^a^^,^ ^c^				√
2-hydroxytetradecane				√
cyclohex-1-en-1-ol		√	√	√
**Other compounds**				
urea^a^^,^ ^b^			√	
2’,6’-dihydroxyacetophenone (2-acetylresorcinol)^c^				√
1-bromo-4-iodo-benzene				√
propane-1,2,3-triol (glycerol)^a^^,^ ^c^	√	√	√	
2-(4-methoxyphenyl)-2-(4-hydroxyphenyl)-propane				√
(E)-3-(4-methoxyphenyl)prop-2-enoic acid (4-methoxycinnamic acid)^c^			√	√
(E)-3-(3-methoxyphenyl)prop-2-enoic acid (3-methoxycinnamic acid)^c^				√
[(2S)-2,3-dihydroxypropyl] (9Z,12Z)-octadeca-9,12-dienoate (1-monolinoleoylglycerol)^a^^,^ ^c^	√	√		
(1R,4aS,10aR)-1,4a-dimethyl-7-propan-2-yl-2,3,4,9,10,10a-hexahydrophenanthrene-1-carboxylic acid (dehydroabietic acid)^c^			√	
Diethylphthalate^b^	√	√		
cyclohexyl, ethyl phthalate				√
17α-ethyl-3β-methoxy-17α-aza-D-homoandrost-5-ene-17-one^a^				√

^a^from an animal source.

^b^contamination.

^c^from a plant source.

## Discussion

The soil in the old city of Jerusalem, which includes the Armenian Garden is predominantly limestone and dolomite with small patches of chalk which break down into terra rosa and pale rendzina. These soils contain oxides of Si, Al, Fe, Ti, Ca, Mg, Mn, Na, K and P [32, 33]. Previous research on soils from tombs around Jerusalem (Matheson unpublished data), identified large quantities of C, O, Si and Ca, that confirms the characterisation of the soil as having high amounts of CaCO_3_ and silicates [34], with very low concentrations of Al, Mg, K, Ti, Fe and S. In addition, Tushingham [31] reports elemental analysis of a flaked piece of soil-covered material from the surface of an iron sword, excavated from the same site. This analysis identified small amounts of S, P, Ar, Cu, Cr, Ni, Sn, Ti and Zr plus a large amount of Si from the depositional environment [31].

There were some compounds that were identified by GC-MS in this analysis that were excluded from the interpretations of each vessel. Some of these are ubiquitous in biological material and in the environment and thus cannot contribute to any interpretation (e.g. urea and lactic acid). There are some compounds that can be from sources of contamination like plasticisers and plastics that were excluded (e.g. phthalic acid, mono(2-ethylhexyl) ester and cyclohexyl, ethyl phthalate). While there are some compounds where a natural source is not known that were also excluded from the interpretation. Consideration has been included in the analysis of the length of time in which these sample have been stored and curated in the ROM. This may have contributed to contamination by plastics and plasticisers, it may have contributed fatty acids from handling and unexplained compounds from dust and aerosol debris. Cellulose based fibres and dust particles were observed under microscopy for all of the removed residue from the ceramic sherds (see S2 File).

The analytical results for the residue recovered off each sherd need to be considered independently because the components identified in each residue are very different. Sherd 737 contained sulphur which can be found in ancient medicines [42–44] and has been documented in the historical literature as a component of thermal, incendiary and explosive materials including “black powder” [45], a mixture of *saltpetre* (potassium nitrate), sulphur and charcoal [46]. The earliest recorded use of sulphur is as an additive to fats and oils to make an incendiary weapon in 429 BC [47, 48]. Sulphur has also been identified in sphero-conical vessels proposed as explosive devices from 12^th^ century Egypt [20, 26]. The residue contained mercury which has been identified in many sphero-conical vessels and interpreted either as part of a medicinal product, the sole contents of the vessel [9, 49] or a residue of the explosive compound mercury fulminate [19]. Mercury has been reported in historical accounts to be a component of incendiary or explosive weapons [45] and many mercury compounds are used in modern explosives [50]. Magnesium was also identified which could have been present in the residue as a pure substance or as magnesium chloride, magnesium sulphate or magnesium nitrate (*nitre of magnesia*). Magnesium chloride is known to have been obtained at very high concentrations in the salt from the Dead Sea [51] and may have been used as a source for magnesium. Nitrates were present in the residue, potentially in the form of calcium nitrate (*norgessaltpetre*), magnesium nitrate, potassium nitrate or sodium nitrate (*soda nitre*). Potassium nitrate has been used in many incendiary and explosive mixtures and was identified by Mercier on sphero-conical vessels [20, 26]. Phosphorus was identified in the residue which could have been present as phosphates or possibly calcium phosphide, another compound suggested as a component of historical incendiary materials [46, 52, 53]. The elevated amount of calcium in the residue will include calcium carbonate from the soil but above the background concentration, may derive from calcium nitrate, calcium phosphide or calcium oxide in the residue. Calcium oxide is reactive in air (to the CO_2_) and water and has been suggested as a component of Greek Fire [46–48, 53, 54]. Lead and iron were identified in this analysis, although they could come from the clay or the residue. Brosh [10] identified iron in a sphero-conical vessel and interpreted it to be pyrite, a material for fire starting. Aluminium was identified in the residue, which could be from the clays used to make the ceramic, if the vessel was produced elsewhere, but given the high levels measured, it is more likely to be present intentionally as part of the vessel contents. Aluminium salts called *alum* have been well known to the ancient Egyptians, Greeks and Romans since at least 3,000 BC [55]. The molecular analysis of the residue from sherd 737 contained a variety of different length fatty acids, most abundant were palmitic acid and stearic acid. These can derive from plant or animal sources [56], although they can also be due to environmental or handling contamination [57, 58]. Some identified fatty acids are found in both plant and animal sources like capric acid, myristic acid and pentadecyl acid. Lauric acid, carballylic acid and salicylic acid have been reported in plants and plant derived natural oils. A monoglyceride, 1-monolinoleoylglycerol, was also identified that derives from both plant oils and animal fat. Glycerides can break down into fatty acids and glycerol. Both fatty acids and glycerol have been identified in the recovered residue (Table 5). Two dicarboxylic acids were identified, suberic acid and azelaic acid. Dicarboxylic acids are predominantly the breakdown products of large fatty acids from plant or animal sources and can also be due to fermentation [59, 60]. Two amino acids were identified in this residue glycine and leucine, however, the presence of these small, simple amino acids only suggest the possibly of a small component of protein. From this residue, 7-methylhexadecane and cetyl alcohol were identified that could indicate the presence of residues from organic materials including plant essential oils, resins, animal fats, beeswax, oil or tar. In summary, the presence of these compounds in the residue from sherd 737, indicate plant oils, glycerol and animal fat. The composition of the active ingredients of some of the earliest thermal weapons include fats, oils, pitch, petroleum, asphalt, bitumen and tar [46, 47]. The results of the molecular analysis are consistent with a container for oils, scented substances (scented oil or perfumes) or medicines as reported by Barnard et al. [21, 27] or a fuel source for a weapon, lamp, or alchemical vessel [45, 50]. It may have multiple uses storing various chemicals or mixtures. However, when considering the elemental and chemical data, the interpretation as a residue from an explosive device, possibly an incendiary or illumination grenade is worth considering further. The thick walls of this vessel would provide the containment and strength to withstand the build-up of pressure prior to detonation, which is required for an explosive weapon [1, 9, 25]. While sherd 737 is from an undecorated vessel with an approximate weight and shape optimal for a grenade [61] and the interpretation of an explosive device is consistent with its recovery from the destruction debris above the medieval surface [31]. Also, the historical accounts, like the siege of Jerusalem in AD 1187, report weapons consistent with grenades thrown against the city by the forces of Saladin [62].

In contrast, the residue removed from sherd 741 is very different due to the absence of elements and the small number of fatty acids. Sulphur is present above the background soil but is also the lowest amount of sulphur detected in the four residues. However, it could still be part of the residue. The molecular analysis identified lauric acid and salicylic acid derived from plant sources; myristic acid from plant or animal sources but is more abundant in plants; and palmitic acid and stearic acid which can come from plant, animal, contamination or the environment. The breakdown compounds azelaic acid and glycerol were identified along with alanine and 1-monolinoleoylglycerol. None of the elemental composition is above the background soil. For residue 741 the analysis is most consistent with the work of Barnard et al. [27] and so the vessel was probably a container for oils, a valuable commodity and consistent with its location in the royal palace.

The analysis of the residue from sherd 742 indicates animal fats, plant oils, resin, salts and mercury. The elemental analysis recorded only a moderate amount of sodium, which could be from added salt, sourced from medicinal or scented salts that were common in the region around the Dead Sea. The high amount of sulphur and mercury mixed with organic material, however, suggests a medicinal product [9, 42–44, 49]. Mercury and sulphur can be present as the mercury sulphide pigment, vermilion. The molecular analysis identified dehydroabietic acid which is found in the resin of a conifer and 4-methoxycinnamic acid which can be found in a wide range of plant exudates. The residue from sherd 742 produced the greatest range of fatty acids (Table 5). There is an abundance of the small fatty acids, levulinic acid, 4-methylvaleric acid and enanthic acid, and although they are not that useful in identifying a source, some have interesting characteristics, for example 4-methylvaleric acid has a cheesy aroma. Other compounds, including levulinic acid and 4-methylvaleric acid, identified in this residue are breakdown products, generated by heating carbohydrates. They can be found in both plant and animal sources but cannot be differentiated from environmental sources. This residue also contained fatty acids from plant sources, including lauric acid, azelaic acid and undecanoic acid. Other compounds identified are usually found in plants and specifically in plant oils, including hexadecanoic acid, 1,1-dimethylethyl ester; salicylic acid, 2-acetylresorcinol and carballylic acid. There were also unsaturated fatty acids including 10-undecynoic acid, oleic acid and palmitoleic acid which are found in plant oils but can be found deposited in animal fat. The compound 10-undecynoic acid has a distinctly woody aroma. There were many other compounds typically found in both plant and animal material like capric acid, tridecylic acid, myristic acid, pentadecylic acid, palmitic acid, margaric acid and stearic acid. The presence of both pentadecylic acid and margaric acid suggests animal fat, most likely from a ruminant [60, 63]. There was cetyl alcohol, glycerol and two dicarboxylic acids (suberic acid and azelaic acid) similar to many of the compounds identified in the residue on sherd 737. Additional hydrocarbons, 2-methylnonadecane and 3,3-diphenyl-1,2-propadiene, were identified which may be contamination or from organic materials including plant essential oils, resins, animal fats, beeswax, oil or tar. The presence of alanine, glycine and urea could indicate the degradation of proteins. The sherd is a fragment of the collar and opening of a vessel. So, the presence of dehydroabietic acid suggests the residue included a resin from a conifer which could have been part of the vessel contents, but given its location may have been used to seal the neck of the vessel, as identified on other archaeological ceramic vessels [64, 65]. The fatty acids described above include a variety of aromatic compounds from plant sources known to provide oily, woody, floral, waxy, cheesy and pine like scent. Plant oils and resins can be flammable, aromatic or bioactive and thus can be found in the archaeological residues of incense [35], scented materials (scented oils, perfume or scented water) [27] and medicinal products [21, 27]. Glycerol is a common solvent used for scented and medicinal products. Overall, the presence in the residue of major components of plant oils, specifically, oleic acid and plamitoleic acid and aromatic compounds strengthens the indication of a vessel used for scented or medicinal products [27]. The GC-MS analysis of sherd 742 is similar to those results obtained by Barnard et al. [27] and Pozhidaev et al. [21] which were interpreted to be a scented product and medicinal product, respectively. However, the extract from sherd 742 also included a high level of sulphur and mercury, which can be found in medicinal materials, so the original vessel was a container for coloured, scented or more likely medicinal material, possibly sealed with conifer resin.

The residue from sherd 744 contains plant oils and animal fats. The compounds identified in this residue include fatty acids, dicarboxylic acids, esters and alcohols. These included plant fatty acids, lauric acid and benzoic acid, and others found in both plants and animals, including myristic acid, pentadecylic acid, palmitic acid, margaric acid and stearic acid. These fatty acids are interpreted to indicate plant oils and animal fats. The animal fat is most likely from a ruminant due to the presence of both pentadecylic acid and margaric acid [60, 63]. The cetyl alcohol, Stearyl alcohol and 2-hydroxytetradecane could be from organic materials including plant essential oils, resins, animal fats, beeswax, oil or tar. Compounds 2-acetylresorcinol, 4-methoxycinnamic acid and 3-methoxycinnamic acid are from plant exudates or oils. The compound 17α-ethyl-3β-methoxy-17α-aza-D-homoandrost-5-ene-17-one is predominantly found in plants. Of particular interest is the wide range of dicarboxylic acids, Adipic acid, azelaic acid and sebacic acid which are primarily due to the breakdown of large fatty acids, but they can also be due to fermentation. Many ancient medications and prescriptions were alcoholic decoctions, infusions or distillates, while others were mixed directly with fermented beverages, such as wine or beer. The elemental analysis shows only a high amount of sulphur. Sulphur has been used in alchemy, ancient medicines [42–44] and ancient weapons [47, 48], but it requires an oxidiser as a weapon, and an oxidiser was not identified in this residue. Therefore, with the absence of any oxidiser, the high amount of sulphur in the residue, the presence of plant oil and animal fat, the possibility of a fermented product and considering the style and decoration of the ceramic, the vessel was possibly a medicinal container.

## Conclusion

Previous research maintains support for a variety of functions for sphero-conical vessels. Based on this analysis, the vessels for each of the four sherds can be interpreted as either a multiple use vessel containing various chemicals or the residue from an explosive material (737); a container for medicinal or scented materials (742); a container for medicinal material (744) and a container for oil (741). The composition of each residue was different, and their main characteristics were compatible with the suggestion that the glazed and decorated sphero-conical vessels would be used to contain valuable commodities. While the small, unglazed, undecorated (or simple etched or incised decorations), highly fired, thick-walled, stoneware vessels may have had a specific function, that did not exist before the 9^th^ century and after the 15^th^ century, implying a defined category with a very unique function, possibly for storage of chemicals (e.g. mercury) or an explosive weapon (e.g. grenade). These differences suggest for the first time, that different ceramic typologies (Vezzoli’s type 1, 2 and 3) may have a similar and very specific use where type 1 is more for chemicals, type 2 for scented material or medicinals and type 3 for oils. For one artefact (742), this research has also identified resin acids from the neck of the vessel, considered to be a component of conifer resin which is likely to have been used to seal the opening. The archaeological context for the excavation site as the royal palace is consistent with the presence of luxury and medicinal products and is also consistent with a sherd of an explosive weapon perhaps used in the historical destruction of the royal palace. Future applications of this analysis of residues to other medieval sphero-conical vessels from a range of archaeological contexts is warranted to characterise the diverse vessel contents and functions, enhancing our knowledge of the production and trade of luxury and medicinal products and alchemy in medieval Middle East.

## Supporting information

S1 File (DOCX)

S2 File (DOCX)
