# Supplementary material for: Composition of trace residues from the contents of 11th–12th century sphero-conical vessels from Jerusalem
Source: PLoS One. 2022 Apr 25;17(4):e0267350. doi: 10.1371/journal.pone.0267350 (PMC9037911; doi:10.1371/journal.pone.0267350)
Supplement: S2 File — (DOCX) [file pone.0267350.s002.docx]

***Gas Chromatography coupled Mass Spectrometry***

A volume of 450 µL of each removed residue solution was transferred to new sterile acid washed 2.0 mL glass autosampler vials and freeze-dried under vacuum for 8 hrs or until dry. Once dried 900 µL of acetonitrile (Sigma-Aldrich) and 100 µL of BSTFA (bis(trimethylsilyl)trifluoroacetamide) with 1% TMS (trimethylchlorosilane) solution (Sigma-Aldrich) was added to each vial, purged with nitrogen and sealed with a teflon-coated septa. The samples were incubated at 120° C for 30 mins (Baxter Scientific Multi-Block) then analysed with GC-MS. The derivatised samples were analysed using a Varion model 450 gas chromatograph coupled with a Varion model 300-MS quadrupole GC-MS mass spectrometer. A factor four capillary column (VF-5ms, 30m x 0.25mm ID, DF=0.25um) was used for the analysis. Helium was used as the carrier gas at a flow rate of 1.0mL/min. Samples were introduced via splitless mode in an autosampler with the injection port at a temperature of 270°C. The column temperature was initially held at 50°C for 2 minutes then increased from 50°C to 155°C at a rate of 8°C/min and then from 155°C to 275°C at a rate of 40°C/min and held at 275°C for 9 minutes. The ionization energy was 70eV and the ion source was set at 200°C under electron ionization (EI) conditions. The scan range was from 40 to 500m/z. The GC/MS interface temperature was set at 266°C. Output files were analysed using Varion MS workstation version 6 and the NIST Mass Spectral Database.

***Microscopy***

The particulate material that remained after all of the volumes of dissolved residue were removed for specific forms of analysis were placed onto microscope slides for microscopic analysis. Using the BX51 Olympus microscope. All of these microscope slides were analysed under bright field and polarized light microscope at 10X, 20X, 40X and 100X magnification objective lenses. The aim for microscopy was to determine the presence of and particulate or microscopic remains that may support the identification of the residue. This could include features like pollen, charcoal, mineral crystals and starch grains. There was nothing of note from the microscopic analysis except some modern cellulose-based fibres consistent with cotton and some uncharacterized particulate material consistent with dust most likely from the curation and storage conditions.


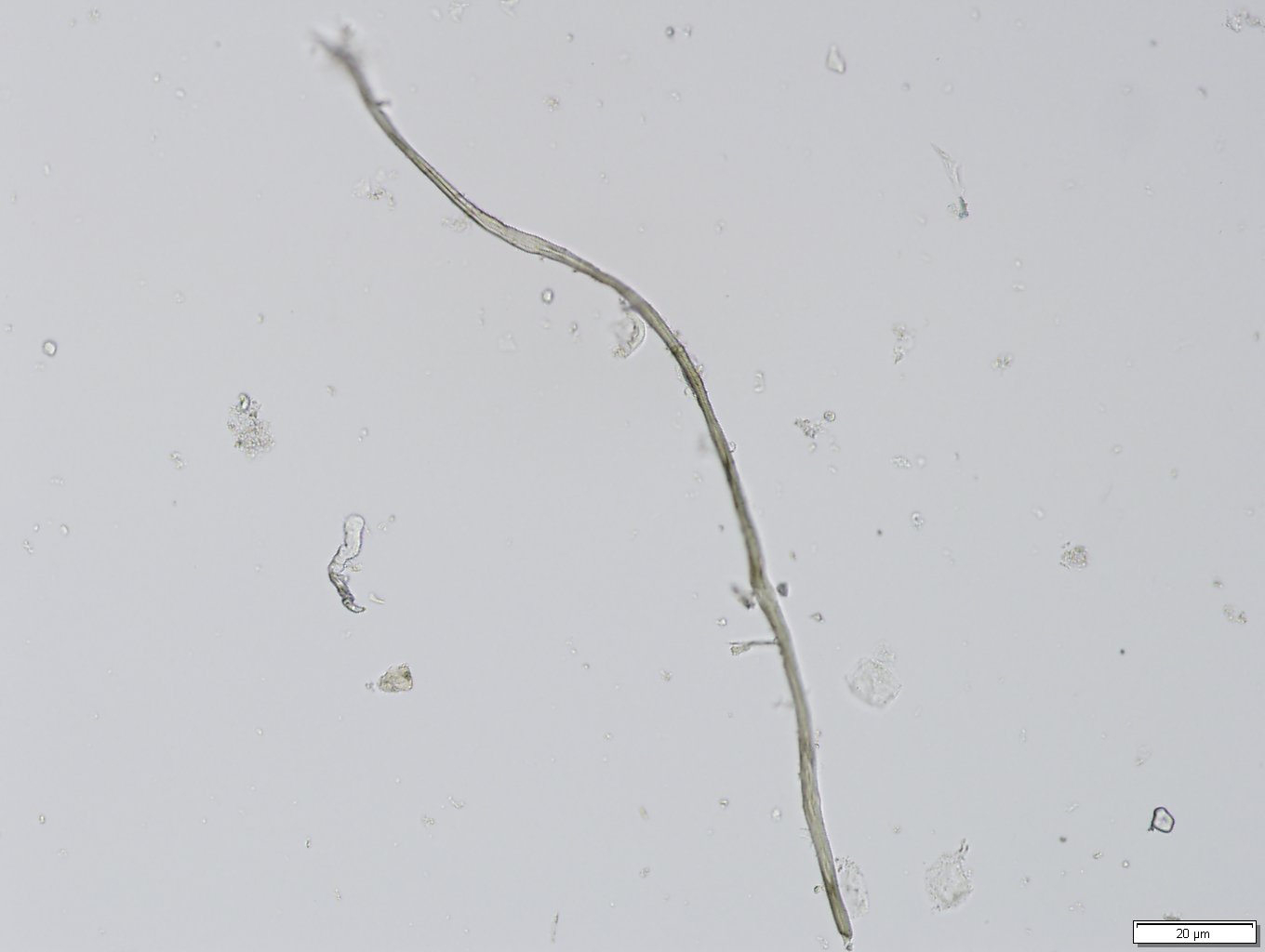


Supplementary Fig. 1. An example of the dust and cellulose based fibres that were identified from the particulate remnants of the residue.
